# Conserved hierarchical gene regulatory networks for drought and cold stress response in *Myrica rubra*

**DOI:** 10.3389/fpls.2023.1155504

**Published:** 2023-04-14

**Authors:** Weijie Xu, Haiying Ren, Xingjiang Qi, Shuwen Zhang, Zheping Yu, Jianbo Xie

**Affiliations:** ^1^State Key Laboratory for Managing Biotic and Chemical Treats to the Quality and Safety of Agro-Products, Institute of Horticulture, Institute of Agro-product Safety and Nutrition, Zhejiang Academy of Agricultural Sciences, Hangzhou, China; ^2^State Key Laboratory of Tree Genetics and Breeding, College of Biological Sciences and Technology, Beijing Forestry University, Beijing, China; ^3^State Key Laboratory for Managing Biotic and Chemical Treats to the Quality and Safety of Agro-products, Hangzhou, China; ^4^Xianghu Lab., Hangzhou, China; ^5^The Tree and Ornamental Plant Breeding and Biotechnology Laboratory of National Forestry and Grassland Administration, Beijing Forestry University, Beijing, China

**Keywords:** Chinese bayberry, ROS scavenging, abiotic stress tolerance, bHLH transcription factor, gene regulatory networks

## Abstract

Stress response in plant is regulated by a large number of genes co-operating in diverse networks that serve multiple adaptive process. To understand how gene regulatory networks (GRNs) modulating abiotic stress responses, we compare the GRNs underlying drought and cold stresses using samples collected at 4 or 6 h intervals within 48 h in Chinese bayberry (*Myrica rubra*). We detected 7,583 and 8,840 differentially expressed genes (DEGs) under drought and cold stress respectively, which might be responsive to environmental stresses. Drought- and cold-responsive GRNs, which have been built according to the timing of transcription under both abiotic stresses, have a conserved trans-regulator and a common regulatory network. In both GRNs, basic helix-loop-helix family transcription factor (bHLH) serve as central nodes. *MrbHLHp10* transcripts exhibited continuous increase in the two abiotic stresses and acts upstream regulator of *ASCORBATE PEROXIDASE* (*APX*) gene. To examine the potential biological functions of *MrbHLH10*, we generated a transgenic *Arabidopsis* plant that constitutively overexpresses the *MrbHLH10* gene. Compared to wild-type (WT) plants, overexpressing transgenic *Arabidopsis* plants maintained higher APX activity and biomass accumulation under drought and cold stress. Consistently, RNAi plants had elevated susceptibility to both stresses. Taken together, these results suggested that *MrbHLH10* mitigates abiotic stresses through the modulation of ROS scavenging.

## Introduction

A disturbance in the environment triggers rapid and global reprogramming of cells, which requires the spatial and temporal coordination of multiple TFs (Zhang et al., 2019; Zhou et al., 2021). Transcriptional regulation occurs on a multitude of time scales, from minutes to days, making temporally dynamic patterns possible (Wang et al., 2021). The regulation of phytohormone signaling pathways, light-signaling pathways, circadian clock regulation, and reactive oxygen species homeostasis at the transcriptional, epigenetic, and post-translational levels have been identified during environmental stress including heat, drought and cold stress (Ohama et al., 2017; Li et al., 2018; Ding et al., 2020).

In the presence of excess ROS, for example due to environmental stress, the cells are subjected to oxidative conditions that are detrimental to them (Wang et al., 2008; Kuge et al., 2010). H_2_O_2_, as ROS, is an important indicator of the generation of ROS (Bhattacharjee, 2012; De la Garma et al., 2015; He et al., 2018). Thus, the maintenance of steady state ROS by scavenging routes is necessary in order to prevent oxidative damage due to adverse environmental stress (Mittler et al., 2004; Miller et al., 2010; Jiang and Jie, 2002). The AsA-GSH cycle and Superoxide Dismutase (SOD) are both essential for scavenging ROS (Shi et al., 2013; Hernández et al., 2015). Superoxide dismutase (SOD) is the primary defense mechanism against ROS in plants, and it converts superoxide to oxygen (O_2_) and H_2_O_2_ at the molecular level (Huang et al., 2016; He et al., 2018; Wang et al., 2021). In the AsA-GSH cycle, APX uses AsA to reduce H_2_O_2_ to H_2_O (Hernández et al., 2015; Xing et al., 2018). In previous studies, APX is present in many organelles as well as in the cytosol. It has a unique ability to adapt to stress in various environments. (He et al., 2018; Xing et al., 2018). For example, it has been shown that overexpression of *APX2* gene increases tolerance to exogenous hydrogen peroxide and assists in ROS detoxification under both stress and normal conditions in *A. thaliana* (Mittler et al., 2006; Rossel et al., 2007).

bHLH superfamily, a TF family with a large number of members, is prevalent throughout eukaryotes (Pires and Dolan, 2010; Sánchez-Pérez et al., 2019; Guo et al., 2021). In general, bHLH is composed of about 60 amino acids and has two functional areas: a base area with 13 to 17 predominantly base amino acids for DNA binding, and an HLH area capable of forming a homodimer or heterodimer with one or more partners. (Tian et al., 2019; Guo et al., 2021). bHLH TFs are important regulators in controlling responses to environmental stresses, development processes (Sun et al., 2020), and the biosynthesis of secondary metabolites (Groszmann et al., 2010; Zhao et al., 2020) by targeting dehydration and cold responsive genes (Guo et al., 2021). In *Arabidopsis*, AtAIB (ABA-inducible bHLH-type transcription factor), which enhance drought tolerance, control stomatal closure by modulating stomatal movement associated with H_2_O_2_ signalling (Cui et al., 2015; Li et al., 2017). In wheat, CBF (ICE1, bHLH116), a MYC-type bHLH TFs, was upregulated by cold stress and the knock-down *bHLH116* seedling displayed reduced cold stress tolerance accompanied with increased ROS levels and reduced antioxidant enzyme activities (Guo et al., 2021). Extensive studies uncovered several downstream genes of *bHLH*. For example, bHLH122, can directly bind to the promoters of *CYP707A3* gene, repressing its expression and increasing the APX content. AtbHLH68, AtbHLH112 and AtbHLH122, have been reported to control abiotic stress responses by regulating the APX and ABA signaling pathway genes in *A. thaliana*. These results indicate that *bHLH* genes play important roles in plant abiotic stress tolerance through crossing with phytohormone ABA and ROS scavenging pathway. However, the functional importance of *bHLH* gene in controlling plant response to multiple abiotic stresses remains to be investigated and the downstream genes extending the bHLH pathway remain unclear.

The Chinese bayberry (*Myrica rubra* Sieb. and Zucc.), which is widespread in tropical and subtropics, is one of the most important sub-tropical fruit crops. It is also a good candidate for studying fruit quality (Ren et al., 2019; Ren et al., 2021). *M. rubra species* are sensitive to drought and cold stress, which will directly decrease the yield and quality of *M. rubra* (Larcheveque et al., 2011; Windt et al., 2011). Moreover, the whole genome of *M. rubra* will be helpful to study the function genome and improve its genetics. (Ren et al., 2019). In this study, we performed a time-course transcriptome investigation in response to drought and cold stress, in an effort to identify the potential multifunctional TFs involved abiotic stress in *M. rubra*.

## Materials and methods

### Plant materials

The 1-year-old *M. rubra* seedlings were grown in a greenhouse at Lanxi, Zhejiang Province, China (29.22 N, 119.45 E) (15.0 h light, 22–25°C, 75% humidity). We watered the seedlings once a day to maintain the optimum level of moisture in the field. In order to carry out the cold-treatment, the plants in the treatment group were transferred to the growing room (Sanyo) where they grew under the same conditions as those of the control (He et al., 2018). Three biological replicates were used for each group of seedlings, with each group exposed to 4°C for 0, 30 min, 1, 3, 6, 9, 12, 24, 36, or 48 h in growth chambers (Sanyo). Treatment groups were moved to Sanyo growth chambers with the same growth conditions as those in the control group when performing drought treatments. The seedlings were also divided into ten groups with three biological replicates each, and seedlings from each group were treated with 8% polyethylene glycol (PEG) for either 0, 30 min, 1, 3, 6, 9, 12, 24, 36 or 48 h in growth chambers (Sanyo) at 23°C. Each of the three to five fully expanded leaves of the seedling were measured.

### Antioxidant enzyme and H_2_O_2_ assay

The pigment content and enzyme activity of *M. rubra* leaves were determined immediately after freezing in liquid nitrogen at the same time. In few words, 0.1 g of the leaf sample was frozen in liquid nitrogen, homogenized in cold 0.01M phosphate buffer (1.5 mL, pH 7.2),and centrifuged at 14,000 grams for 10 minutes at 4°C for 10 minutes, and then Plant superoxide dismutase (SOD) Assay Kit (Nanjing Jiancheng Bioengineering Institute, Jiangsu Province, China) was used to measure the activity of SOD. First, 1.5 mL reaction buffer (6.5 × 10^-6^ M riboflavin, 0.013 M met, 6.3 × 10^-6^ M NBT, 1 × 10^-4^ M Ethylene Diamine Tetraacetic Acid (EDTA), 0.05 M phosphate buffer, pH=7.8) was added to the supernatant followed by incubation at 25°C for 30 min, and absorbance at 560 nm was measured with a spectrophotometer. The activity of malondialdehyde (MDA) was measured in accordance with the manufacturer’s specification using Catalase Assay Kit (Nanjing Jiancheng Bioengineering Institute). Using a plastic pestle and an ice-cold 0.01 M phosphate buffer (pH 7.2) with 1.13 mg dithiothreitol, the leaf specimens were crushed in a micro-centrifuge. A centrifuge was used for 10 minutes at 4°C, weighing 14,000 g. We assayed the supernatant for MDA activity by measuring the linear rate of decrease in absorbance at 240 nm with a spectrophotometer. For APX and peroxidase (POD) activity, 0.1 g samples of leaves were homogenized in 1.5 mL ice-cold 0.01 M phosphate buffer (pH 7.2) for 30 min and centrifuged at 14,000 g for 10 min at 4°C. A Plant APX and POD Assay Kit (Nanjing Jiancheng Bioengineering Institute) were used to measure APX and POD activity in the supernatant. The supernatant was added to a mixture of 0.5 mL 0.1 M phosphate buffer, 0.5 mL 0.1 M guaiacol buffer and incubated at 30°C for 8 min. The absorbance of the sample at 470 nm was measured with an optical spectrophotometer.

### RNA-sequencing and data analysis

In the case of drought and cold-stress, the 3rd to 5th leaves of M. rubra were picked up, then frozen in liquid nitrogen, and kept at -80 degrees Celsius until application. According to the manufacturer’s guidelines, RNeasy Kit (Qiagen) was used to extract the total RNA. NanoDrop ND-2000 (A260/A280 1.9-2.1) and Agilent 2100 bioanalyzer (28S/18S 1.8-2.0) were used for the determination of RNA quality. A strand-specific RNA-seq library was constructed on an Illumina HiSeq 4000 platform according to the manufacturer’s instructions and index codes. The Beijing Novogene Technologies performed the construction of the libraries and the paired-end sequencing. Following quality control and removal of adapter-and poly(N)-containing reads, clean reads acquired after mapping on the reference genome of *M. rubra* (http://www.bayberrybase.cn/) were analyzed (http://www.bayberrybase.cn/) as previously described (Ren et al., 2019) with TopHat (v. 2.0.0) with default parameters (Trapnell et al., 2009). Transcript levels were normalized based on FPKM with Cufflinks (v. 2.1.1) with default options (Trapnell et al., 2012). It was considered significant that genes with *P-*value<0.05 (adjusted for the false discovery rate, Q*-*value< 0.05) and >2-fold change were differentially expressed.

### Functional enrichment analysis and visualization

A GO analysis was performed on DEGs and a GO annotation was obtained from *M. rubra* (http://www.bayberrybase.cn/) (Ren et al., 2019). The result of the GO enrichment analysis along with the *P*-value modified using the Benjamini and Hochberg (1995) FDR method were input into OmicShare platform (https://www.omicshare.com/tools), and created a visual tree map of the outcome of the GO analysis. Significant enrichment of GO terms with corrected *P* value < 0.05 and *Q* value < 0.05 was observed.

### Measurement of tissue specificity

The tissue specificity score was calculated as previously described (Liao and Zhang, 2006). The tissue specificity score was generated as follows in order to further quantify the tissue specificity of gene expression: n represents the sum of tissues, aij is the mean expression of the gene i in tissue j, and the tissue specificity of the gene i is defined as:


$$T\text{i}=\frac{1}{n-1}\sum_{j=1}^{n}(1-\frac{aij}{(aij)\max j})$$

### Construction of a multi-layered hierarchical gene regulatory network using the BWERF algorithm

Using PlantPAN v.2.0, it was determined that cis-regulatory elements were found in the 2 kb promoter region of the candidate genes (Chow et al., 2016; Xu et al., 2022). It was predicted that these motifs are TF target sites based on 80% confidence values. Then, a backward elimination random forest (BWERF) algorithm is employed to construct ML-hGRNs, which uses the genes and TFs encoding transport, photosynthesis and oxidoreductases (Deng et al., 2017; Xu et al., 2022).

### Phylogenetic and bioinformatics analyses

ExPASy (http://web.expasy.org/computepi/) was used for the analysis of the isoelectric point (pI) and the molecular weight of the bHLH protein (Kumar et al., 2018). In order to analyze genetic variation and phylogenetic relationships, a plurality of sequence alignment of bHLH protein sequences was performed in MEGA 7 (Kumar et al., 2018). To deal with the gaps and lack of data, we chose a partial deletion with an 80% coverage limit. Therefore, Jones-Taylor-Thornton (JTT) + (G) + (F) was chosen as the optimum amino acid replacement model. The Maximum Likelihood (ML) method in MEGA 7 was utilized to construct a phylogenetic tree of protein sequences with 1000 bootstrapping replicas (Kumar et al., 2022). For all positions, 90 per cent of the site coverage was removed; in other words, no more than 10 per cent of the alignment space, no data, and no clear basis were permitted. A phylogenetic tree was visualized using Figtree software (http://tree.bio.ed.ac.uk/software/figtree/).

### Plasmid construction and *A. thaliana* genetic transformation

Generic transformation, cloning and expression analysis were carried out on *A. thaliana* seedlings at long time (16 hours/8 hours darkness) to produce overexpression and RNAi lines (Quan et al., 2021; Zhang et al., 2022). With the help of gene-specific primers, we cloned the full-length coding region of *MrbHLH10* gene from a *M. rubra* clone template cDNA. During the process of cloning the full-length gene, the coding region of *MrbHLH10* gene was cloned into pDONR222 vector. Subsequently, LR reactions were employed to re-sequence the encoding area and target vector pGWB405 in accordance with Nakagawa et al. (2007), verified by sequencing. Two kinds of vector were introduced to *Agrobacterium* GV3101 *via Agrobacterium*-mediated transformation together with gene silencing inhibitor P19, and then transformed into *Nicotiana benthamiana* based on previous study (Zhang et al., 2022). The *Agrobacterium*-mediated floral dipping approach was used to create transgenic *A. thaliana* seedlings (Wang et al., 2022). Transgenic plants were confirmed by PCR analysis using vector- and gene-specific primers in **Table S1**.

### Measurement of the physiological characteristics of transgenic plants

On a plate containing 1% sucrose and ½×MS medium (0.1 mM MgSO_4_, 0.1 mM CaCl_2_, 0.6 mM NaCl, and 0.3 mM ZnSO_4_), seeds of WT and transgenic *A. thaliana* were grown. The germination count was calculated up to the 5 days (d) after stratification (nearly emerged radicle). Based on the prior research, we obtained the germination rate (GR) by the number of seeds on each side of WT *A. thaliana* and transgenic plants (Quan et al., 2020; Zhang et al., 2022). Leaf physiological traits were measured in mature leaves of WT and transgenic *A. thaliana* seedlings at 20 days after germination (DAG). Seedling length of *A. thaliana* was calculated by ImageJ software (https://imagej.en.softonic.com/) (Banugopan et al., 2012).

### Statistical analyses

The statistical significance of treatment differences was assessed with either one way or twoway analysis of variance, with SPSS 17.0 (IBM, Chicago, IL, USA) and Excel 2013 (Microsoft Corp., Redmond, WA) on the basis of prior research (Xu et al., 2022). For the calculation of *P* values, the Students’t test (* *P* < 0.05, * * *P* < 0.01) was adopted. After normalization, all samples had a normal distribution with respect to variance homogeneity (Xu et al., 2022).

## Results

### Physiological and transcriptomic changes response to drought and cold stress

**Figure 1:**
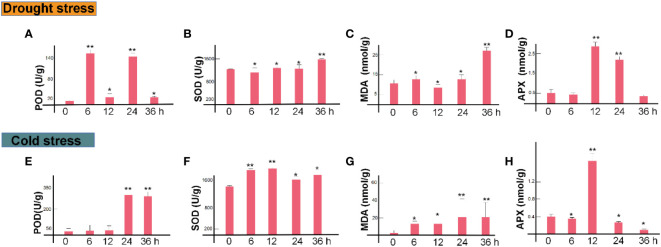
Temporal dynamics of *M. rubra* physiological characteristics during drought and cold treatment. Peroxidase (POD) activity **(A)**, superoxide dismutase (SOD) activity **(B)**, MDA **(C)** and APX content **(D)**, in *M. rubra* under drought stress. Peroxidase (POD) activity **(E)**, superoxide dismutase (SOD) activity **(F)**, MDA content **(G)** and APX content **(H)**, in *M. rubra* under cold stress. (*P < 0.05, **P < 0.01, Student’s t-test).

In order to determine the effects of drought and cold stress on physiological activity, we first examined the activity of SOD, POD, MDA, APX, CAT and H_2_O_2_ under drought and cold stress. As expected, the activity of SOD, POD, MDA, APX, CAT and H_2_O_2_ showed an overall increase during the drought and cold treatment (**Figures 1A–H**). For example, under drought stress, the activities of SOD, MDA, APX and CAT and H_2_O_2_ increased significantly from 6 to 36 h (**Figures 1B–D**); the activities of SOD, MDA and APX peaked at 36 h, 36 h and 12 h, respectively (**Figures 1B, C**). By contrast, the activities of SOD and APX peaked at 12 h after cold treatment (**Figures 1B, D**). Interestingly, the activities of APX and MDA were positively correlated with each other (*P*>0.05, *R*>0.6) under the two stresses. Totally, the anti-oxidant enzyme activities were strongly induced under drought and cold stress.

**Figure 2:**
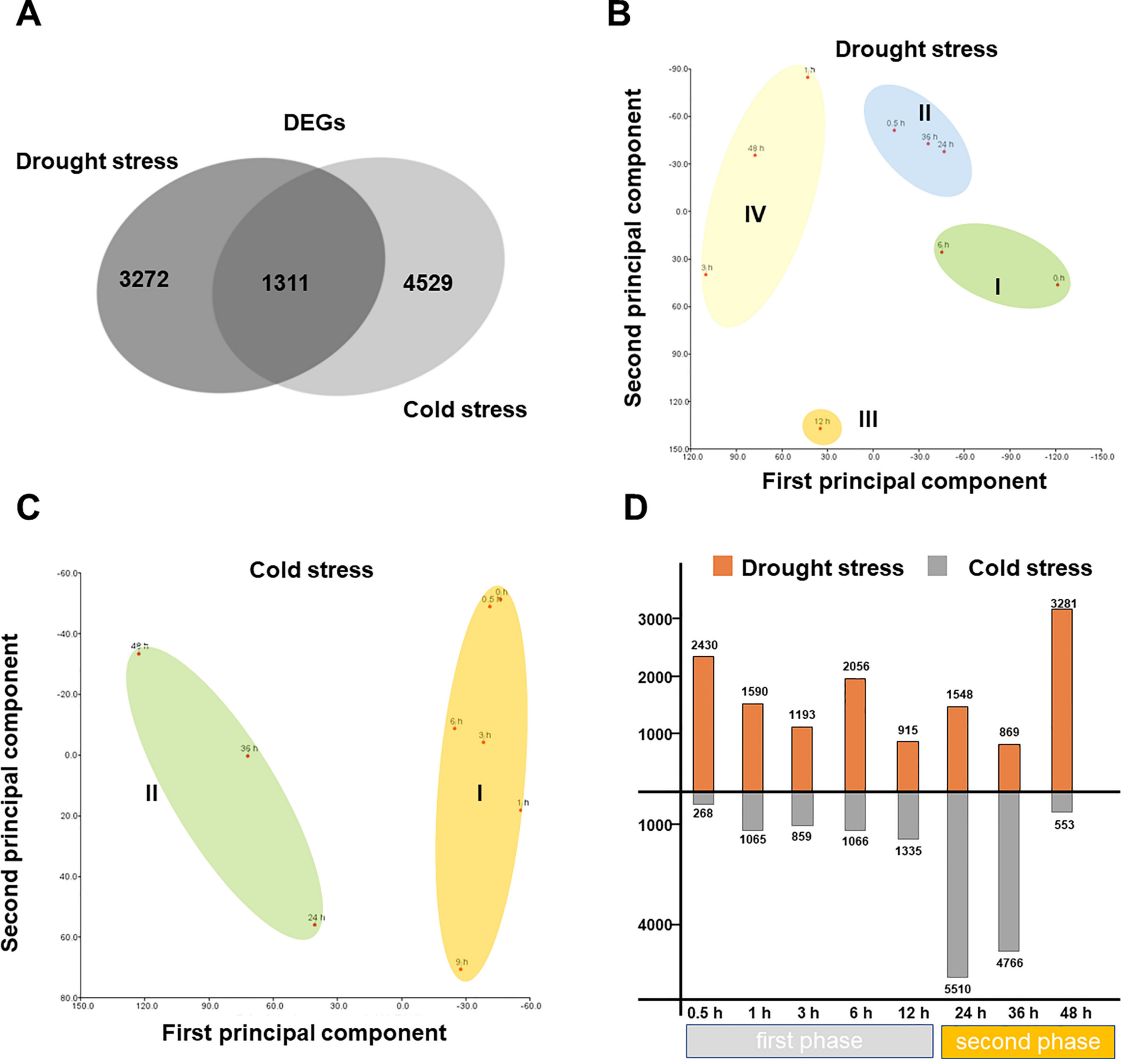
Temporal dynamics of *M. rubra* transcriptome during drought and cold treatment. **(A)** Venn diagrams of DEGs overlapping between drought stress and cold stress. DEG indicates dfferentially expressed gene. **(B)** PCA of the transcriptomes of the 8 time point samples under drought stress and **(C)** cold stress. **(D)** Number of drought-responsive (upper bars) and cold-responsive (lower bars) genes at each time point compared to the control group (0 h).

To identify the stress-responsive genes under both stresses, we sequenced the total RNAs of the leaf tissues across all the time points. A total of 7,583 and 8,840 differentially expressed genes (DEGs) were obtained under drought stress and cold stress, respectively (Fold Change>2 and FDR < 0.01) (**Figure 2A**; **Table S1**). To gain insight into the transcriptome dynamics under the two stresses, we performed principal component analysis (PCA; **Figures 2B, C**). As a result, the transcriptome data can be generally divided into four and two clusters under drought and cold stress, respectively (**Figures 2B, C**). Consistent with this, a previous study reported that abiotic stress response in higher plants occurs in different phases (Wu et al., 2021). Notably, the cold-responsive transcription profile was clearly divided into two phases, and the majority of cold-responsive DEGs were induced in the second phase timepoint from 24 to 48 h (**Figure 2D**). By contrast, there were only <1500 DEGs in the first phase (0 to 24 h. The fewer DEGs within the early response might be reminiscent of *M. rubra* species that lived in freezing environment (under 4°C) with consistent cold pressure (Ren et al., 2019).

The GO analysis of the drought and cold-response genes showed that there were 251 and 227 significant enrichment terms (*P*<0.001). The upregulated DEGs under drought stress were enriched for terms including “response to hormone-mediated signaling pathway,” “oxidoreductase activity,” “photosynthesis,” “transcription factor activity,” “abscisic acid biosynthetic process,” and “ABA signal transduction pathway” (**Table S3**). By contrast, upregulated cold-responsive DEGs were enriched for terms of photosynthesis, response to “oxidoreductase activity,” “photosynthesis,” “external biotic stimulus,” “transcription factor activity,” “transcription regulation,” “calcium-binding,” “DNA binding,” and “serine/threonine kinase activity” (**Table S3**). These indicates that the patterns of the functional shifts were consistent with the physiological changes under the two stresses.

### Conserved and divergent dynamic transcription profile between the two abiotic stresses

**Figure 3:**
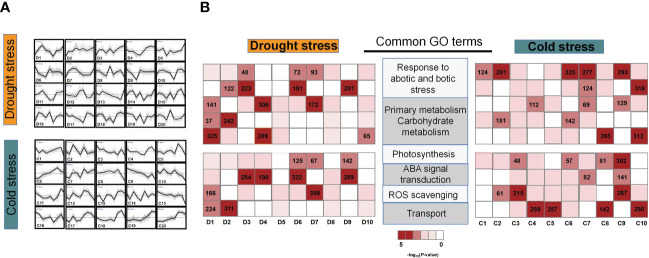
Temporal dynamics of *M. rubra* transcriptome during drought and cold treatment. **(A)** Expression dynamics of the 20 largest gene clusters over time under drought (D1–D20) and cold stress (C1–C20). **(B)** GO terms enriched in DEGs, with red indicating more significant enrichment (–log10(*P*-value)). The heatmaps show enriched GO terms detected in temporally dynamic groups of both drought and cold stress from 1 to 48 h.

To examine the shared and unique transcriptomic network under drought and cold stresses, DEGs were clustered into 20 dynamic groups by using K-means clustering algorithm (Wu et al, 2021). The ten largest dynamic groups in response to drought and cold stress contained most of the stress-responsive DEGs—83.4% (3,142 drought-responsive genes) and 88.7% (4,108 cold-responsive genes) respectively (**Figure 3A**). The genes in the D2, D3, C3, C9, and C10 groups were continuously down- or up-regulated under both stresses (**Figure 3A**). On the other hand, the D1 and C2 groups were observed to have transient changes in gene expression at earlier timepoints, presumably in response to early stress. Thus, the groups with similar expression dynamics probably kept similar biological processes regardless of the stress they were exposed to. Under the two types of stress, the D4 and C4 groups with transient gene expression peaks at 36 hours showed a higher level of photosynthetic gene accumulation (**Figures 3A, B**). Likewise, the D3 and C7 groups that showed an increasing tendency to continue showed oxidoreductase activity, as well as the genes associated with abiotic stress reaction (**Figures 3A, B**). These GO terms are consistent with the early and late physiological changes, which were observed in plant cells under drought and cold stress (Muns et al., 2007; Guo et al., 2021). This similarity in biological functions indicates that abiotic stress-response genes have maintained conserved expression dynamics.

Our objective was to gain insight into the regulatory mechanism underpinning drought and cold stress by using hierarchical clustering (**Figures S1**, **S2**). Under drought and cold stress response, the hierarchical clustering network consist of differentially expressed genes with 6 cluster (DI-VI) and 4 cluster (CI-IV) (**Figures S1**, **S2**), respectively. Cluster enrichment analyses revealed that, despite the presence of multiple metabolic pathways, some patterns could be discerned in a single cluster. For example, in drought stress response, genes involved in “membrane,” “abiotic stress stimulus,” “oxidoreductase activity,” “response to abiotic stress,” “response to stress,” “ADP metabolic process,” and “oxidation-reduction process,” were mainly enriched in cluster D-III, which exhibited an increasing trend from 12 to 36 h, indicating that membrane biosynthesis, ADP metabolic process and oxidoreductase activity pathways are enhanced in response to drought stress (**Figure S1**). In cold stress response, genes involved in “oxidoreductase activity,” “abiotic stress stimulus,” “antioxidant activity,” “photosynthesis,” and “plant-pathogen interactions” were enriched in cluster C-IV and exhibited an increasing trend from 0 to 12 h, suggesting active ROS scavenging. DEGs involved in “response to abiotic stimulus” and “organic substance biosynthetic process,” and “response to cold,” were enriched in cluster C-II and showed continuously decreasing trends to 48 h (**Figure S2**). As a result of drought and cold stress, *M. rubra* exhibits early and late physiological changes that are consistent with these two GO terms.

### Transcriptional regulatory networks involved in drought and cold stress

**Figure 4:**
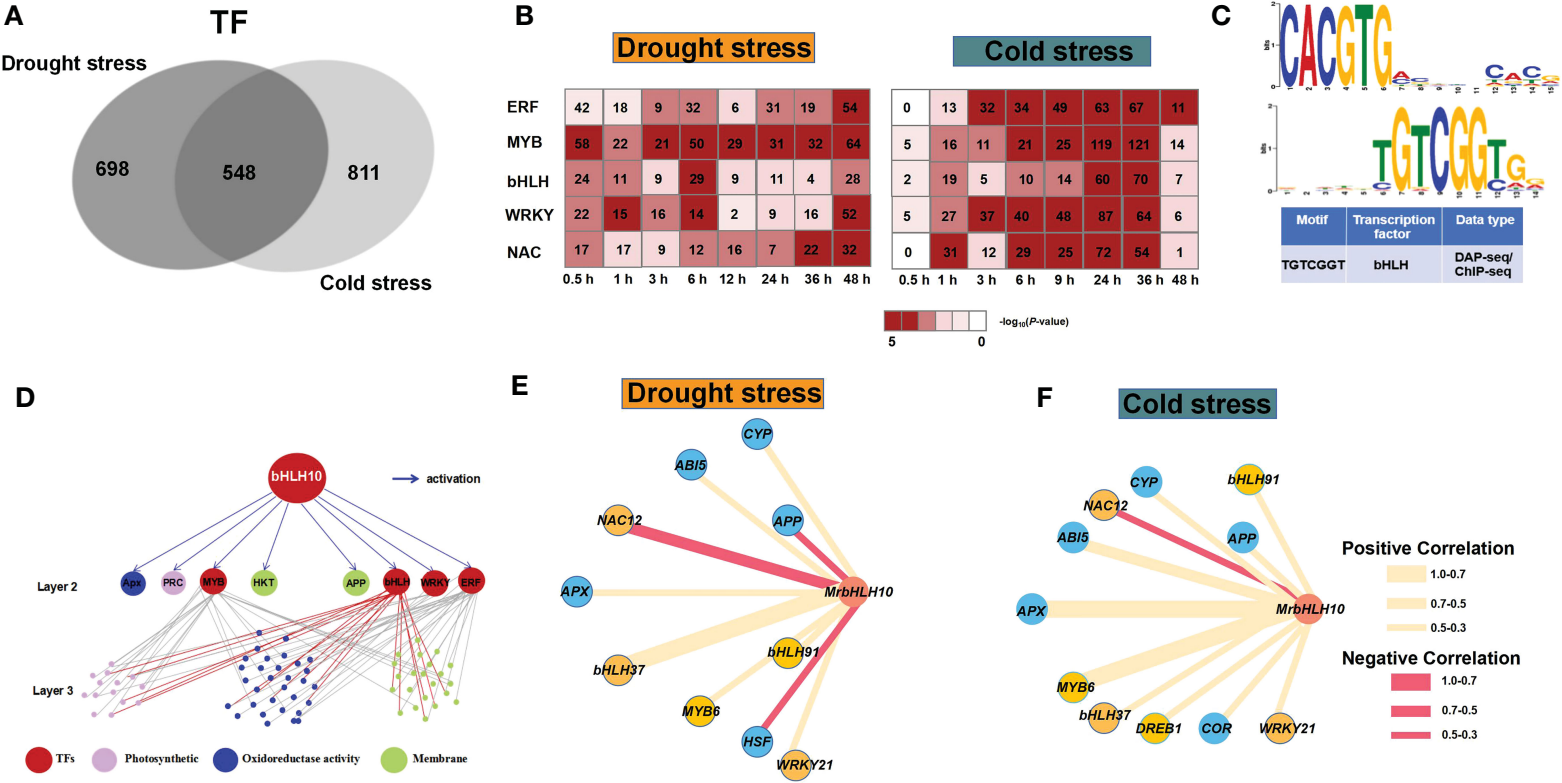
Gene regulatory networks of drought and cold-responsive transcriptional modulators. **(A)** Venn diagrams of TFs overlapping between drought stress and cold stress. **(B)** Enrichment (–log10 (P-value)) of TF families in timepoint (0–48 h) under drought stress (left) and cold stress (right). The number represents the number of differentially expressed TF genes. The heatmap presents TF families enriched in at least one of 20 groups. **(C)** Bioinformatic analysis of bHLH Binding Site (TFBS) Motifs based on PlantTFDB database (http://planttfdb.gao-lab.org/). **(D)** Gene regulatory network of photosynthesis, oxidoreductase activity and membrane regulation under drought stress and cold stress. **(E)** Coexpression network of genes that are differentially expressed under drought stress and cold stress **(F)** treatment in *M. rubra* at different timepoints.

Expression dynamics are conserved, indicating that some regulators are common to both early and late responses in two abiotic stresses (Wu et al., 2021). Gene expression profiles under abiotic stress were sample-specific for TFs, which dominated the network rather than other genes (Yin et al., 2019; Wu et al., 2021). In our current research, a total of 1,923 and 1,731 stress-responsive TFs were identified under drought and cold stress, respectively, 26.6% (548) of which are stress-conserved TFs (**Figure 4A**). TFs had more sample-specific expression profiles under different abiotic stresses (Wu et al., 2021), and the number of stress-responsive TFs increased significantly across the samples in our study (**Figure 4B**). Specially, TF families of bHLH, MYB, ethylene responsive factor (ERF), MYB, WRKY, and NAM, ATAF, and CUC (NAC) showed one of the highest enrichments under drought and cold stress (**Figure 4B**). The bHLHs proteins, for instance, form a large family of plant-specific TFs that play an important role in plant defense and biotic and abiotic stresses (Yoda et al., 2002; Journot-Catalino et al., 2006; Zheng et al., 2006; Liu et al., 2007). Interestingly, the transcription of *MrbHLH18*, *MrbHLH31*, *MrbHLH10* and *MrbHLH75* increased significantly under both drought and cold stress, which are grouped in cluster D3 and C7 related to response to abiotic stimulus and oxidoreductase activity in both abiotic stresses.

To further examine the potential regulatory relationships, we constructed gene regulatory network (GRN) that interlinks TFs with their potential target genes (PTGs) based on the expression data and examined the presence of *MrbHLH10* potential TF binding site (TFBS) to verify the GRN (**Figure 4C**). As a result, totally 227 TF-PTG pairs were identified, and we observed that MYBs, WRKYs, bHLHs and ERFs which targeted large numbers of genes (twenty or more target genes from each TF as indicated by solid or arrows line, **Figure 4D**). Notably, *MrbHLH10* regulated 39 downstream ROS signaling pathway genes, and clear functional associations were observed in both stresses—*MrbHLH10* were connected to *APX* (GENE_006840) gene (**Figures 4E, F**; *R* > 0.6; *P <*0.05), while multiple previously stress-responsive gene, such as *WRKY21* (Zhang et al., 2017) and *MYB6* (Li et al., 2020) were also associated with *MrbHLH10* (**Figures 4E**), indicating a role as the master regulators under the cold and drought stress.

### Molecular characterization of MrbHLH10

**Figure 5:**
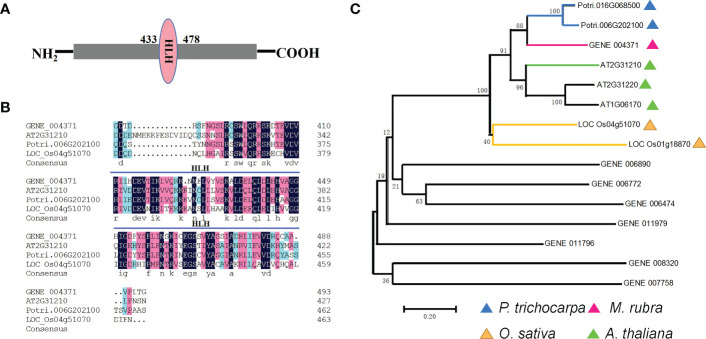
Bioinformatics analysis of *bHLH* gene **(A)** Domain architecture analysis of MrbHLH protein. **(B)** Multiple amino acid sequence alignment of bHLH10 from *M. rubra*, *O. sativa*, *P. trichocarpa* and *A*. *thaliana*. **(C)** Phylogenetic analysis of bHLH homologs from *M. rubra*, *O. sativa*, *P. trichocarpa* and *A*. *thaliana*. Scale bars, 0.2.

The bHLH superfamily, one of the largest TF families, is widespread in eukaryotes (Pires & Dolan, 2010). Among the amino acid sequences of the MrbHLH proteins, there is a high conservation domain comprising 60 amino acids with two distinct functional areas (**Figure 5A**). The base region, which is located at the N-terminal end of the domain, participates in DNA binding, is composed of 15 amino acids with many base residues (Tian et al., 2019). The MrbHLH10 coding sequencing (CDS) is 1,483 bp long, encoding 493 amino acids and having a molecular mass of ~54 kD and an isoelectric point of 5.42 (**Table S6**). The multiple amino acid sequence alignment and a phylogenetic tree indicated that the bHLH protein sequences from multiple species consist of the same conserved domains (Guo et al., 2021) (**Figures 5B, C**). Therefore, MrbHLH10 and ATbHLH10 might have the same biological function, which has been demonstrated to respond to various abiotic stresses (Guo et al., 2021).

In order to probe into the potential biology function of *MrbHLH10* gene in *M. rubra*, we made a deep analysis on the expression mode of bHLH. Notably, a total of 241 *MrbHLH* members are detected in *M. rubra*, which show four major expression patterns (**Figure S3**). MrbHLH10 showed different expression patterns in 12 of the selected organs or tissues (**Figure S3**), for instance, the expression profiles of *bHLH10* gene in the branch, xylem and mature leaf show similar trends, and expression patterns in the young leaf, phloem and petiole grouped in one cluster (**Figure S3**), indicating that *bHLH10* gene regulate plant growth and stress response in *M. rubra*.

### MrbHLH10 promotes APX and ROS scavenging in response to environmental stresses

**Figure 6:**
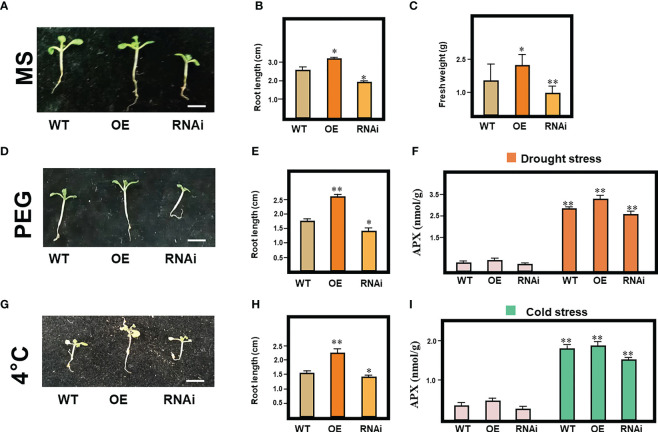
*MrbHLH10* expression in response to drought and cold stress. **(A)** Time course of *MrbHLH10* expression in *A*. *thaliana* exposed to drought and cold stress on standard ½ MS medium. **(B)** Root length and **(C)** fresh weight of wild-type (WT), *MrbHLH10*-OE and *MrbHLH10*-RNAi lines grown on standard ½ MS medium for 1 weeks. **(D)** Time course of *MrbHLH10* expression in *A*. *thaliana* exposed to drought stress on standard ½ MS medium with 8% PEG for 1 weeks. **(E)** Root length of wild-type (WT), *MrbHLH10*-OE and *MrbHLH10*-RNAi lines grown on standard ½ MS medium with 8% PEG for 1 weeks. **(F)** APX content in leaves of wild-type (WT), *MrbHLH10*-OE and *MrbHLH10*-RNAi lines grown on standard ½ MS medium with 8% PEG for 1 weeks. **(G)** Time course of *MrbHLH10* expression in *A*. *thaliana* exposed to drought stress on standard ½ MS medium with 4 °C for 1 weeks. **(H)** Root length of wild-type (WT), *MrbHLH10*-OE and *MrbHLH10*-RNAi lines grown on standard ½ MS medium, with 4 °C for 1 weeks. **(I)** APX content of wild-type (WT), *MrbHLH10*-OE and *MrbHLH10*-RNAi lines grown on standard ½ MS medium, with 4 °C for 1 weeks. Data are presented as means ± SD (n = 3). (*P < 0.05, **P < 0.01, Student’s t-test).

To investigate the potential function of bHLH10 in the response to enviromental stresses, we generated transgenic *A. thaliana* seedling overexpressing (OE) and RNAi bHLH10 *via Agrobacterium tumefaciens*-mediated transformation (**Figure 6A**) (Zhang et al., 2022). Compared to the WT and RNAi line, the OE line had a significantly longer root length and heavier fresh weight increased by 22.9% and 62.8% respectively under normal condition (*P*<0.05; **Figure S3**), indicating that *MrbHLH10* gene is implicated in plant growth (**Figures 6A–C**).

Compared with WT and RNAi lines, the primary root length of OE was obviously increased (*P*<0.05; **Figure 6D**). After 7 days of treatment at 4 °C, the WT and RNAi lines showed a significant increase in the primary root length of OE line compared with WT and RNAi (*P*<0.05; **Figure 6G**), indicating that *bHLH10* can positively increase the growth of plants under drought and cold stress. Ascorbate peroxidase (APX) maintain cellular ROS homeostasis and is major ROS scavengers (Xing et al., 2018; Wang et al., 2021). APX activity were measured in OE, RNAi and WT line. Total APX activity was strongly induced, and OE line showed distinctly APX activity higher than WT plants and RNAi line respectively under drought and cold stress (*P*<0.05; **Figures 6F, I**). Thus, bHLH10 could be used as an indirect way to improve drought and cold-tolerance by keeping ROS in a stable state.

## Discussion

### Presence of conserved gene regulatory network in response to environmental stresses

The improvement of the plant’s ability to withstand many kinds of stress is one of the fundamental objectives of breeding. It is of great importance to enhance the comprehensive resistance of plants by means of core functional genes in molecular breeding. (Hu et al., 2017; Xu et al., 2023). In this study, we constructed a high-temporal-resolution dynamic transcriptome landscape of drought and cold stress responses using nine time points. The dynamic transcriptome profiles were clealy grouped into four and two stages within the drought and cold stress response respectively, indicating the early-responsive and late-responsive phase (Hickman, 2017; Wu et al., 2021). We found there were 7,453 and 9,614 genes mainly expressed at the stages of early- and late-responsive phase respectively. Biological processes in the K-Means groups with similar expression dynamics were probably maintained across the two types of abiotic stresses. It is also possible that there are common regulators of early and late responses in both stressors due to the conserved expression dynamics. Specifically, we have identified 2,311 stress-conserved genes, among them 548 TFs, which will undoubtedly become the goal of functional genomics in the future. Because of this vast array of genes, we will be able to carry out further functional research, which will significantly improve our knowledge of the genetic mechanisms that govern the response to environmental stresses.

The TFs of bHLH, MYB, NAC, WRKY and ERF are known to regulate stress-responsive genes (He et al., 2018; Wu et al., 2021). Some TFBSs, which were enriched in the promoter, were related to the up-regulation of TF genes. The *bHLH* TF gene and some bHLH binding sites were significantly enriched (Guo et al., 2019; Yu et al., 2021). Therefore, transcriptomic regulation during salt stress in *M. rubra* is mediated by dynamic regulation of bHLHs and other TFs. In *A. thaliana*, these TFs are also associated with a cold stress response (Xu et al., 2014; Guo et al., 2021; Yu et al., 2021). In addition, *bHLH* genes function in leaf formation and growth as well as heat and drought stress responses (Khadiza et al., 2017; Zhao et al., 2021), indicating that in the two main dicotyledons and probably in other plants, bHLH orthologues are thought to confer a stress-tolerance mechanism.

### bHLH is implicated in environmental stresses tolerance and plant growth

To adapt to abiotic stress conditions including extreme temperatures (heat and freezing), plants regulate a series of genes, so as to form a GRN in previous study (Guo et al., 2021; Jia et al., 2022). In the absence of a central trans-regulator gene, a GRN could be deleted completely or network connectivity could be reorganized (Wu et al., 2021; Xu et al., 2022). However, there is still little investigation on the construction of GRNs in response to abiotic stresses combination (Reményi et al., 2004; Song et al., 2016; Vihervaara et al., 2018).

Systemic signaling in plants during abiotic stress combination (Zandalinas et al., 2020). In the bHLH10-mediated GRN, direct target genes including MYB6, WRKY41, TCP4, CYP1, STZ and APX1 were identified. In *O. sativa*, overexpression of *OsbHLH148* gene increases the plant’s drought tolerance by regulating the JA pathway and the expression of OsJAZ protein (jasmonate ZIM domain) (Seo et al., 2011). With the expression of *MfbHLH38* gene from resurrection plants (*Myrothamnus flabellifolius*), transgenic *Arabidopsis* has improved water retention and drought tolerance, as well as the increase of their oxidative stress tolerance and osmotic regulatory ability, which is associated with the ABA response and elevated ABA content. In our study, multiple target genes of bHLH10, such as APX1 and WRKY41 were known to function in ROS scavenging and ABA signal processes (Reményi et al., 2004; Song et al., 2016; Vihervaara et al., 2018). In addition, recent studies showed that AtSTZ, a downstream transcription repressor, enhances abiotic stress tolerance after growth delay in *Arabidopsis* (Park et al., 2015; Sakamoto, 2004), may be regulated by MAP kinases in *Arabidopsis* (Nguyen et al., 2016). CYP1 mediate the last steps of auxin biosynthesis, as well as root growth inhibition in response to stress (Rodríguez et al., 2010).

The bHLH TFs are also involved in the course of development and growth, including the germination of seeds and the development of root, epidermis, xylem, carpels, anthers, fruits and stomata (Groszmann et al., 2010; Guo et al., 2021). In our study, root length significantly increased 131%, 114% and 78% in the *MrbHLH10*-OE line compared to the WT under drought, cold and normal condition respectively. Currently, there is evidence that bHLHs directly regulate cytokinin synthesis genes or cytokinin degradation genes such as CKXs (Hezhong et al., 2008; Hozain et al., 2012). bHLH10 may directly regulate *MrCKX* by GRN analysis, enhancing root development and cell division in root of OE plants, in agreement with prior reports (Hezhong et al., 2008). In contrast to other stress-tolerant genes like MYB6, DREB2C, WRKY45, and AtSAP5 (Lim et al., 2007; Hozain et al., 2012), overexpression of *bHLH10* gene enhances plant growth and environmental stresses tolerance.

### *MrAPX1* may be a key downstream gene involved in ROS scavenging

Excess ROS, for example, caused by environmental stresses, such as heat, salt, cold, and drought stress, results in oxidative conditions that are detrimental to plant cells (Wang et al., 2008; He et al., 2018). In response to environmental stresses, plants accumulate cryoprotectant molecules such as soluble sugars, sugar alcohols, and low-molecular-weight nitrogenous compounds (proline and glycinebetaine; Zhuo et al., 2017; He et al., 2018; Xing et al., 2018), and activated antioxidant defence systems which include MDA, CAT, SOD, POD and APX (He et al., 2018; Wang et al., 2021; Xie et al., 2018; Zhuo et al., 2017). These antioxidative enzymes can suppress ROS accumulation in plant cells due to environmental stress (Wang et al., 2021; Wu et al., 2018). The cycle of AsA-GSH and SOD is of great significance to the scavenging of ROS (He et al., 2018; Shi et al., 2014). SOD acts as a first defence mechanism for ROS, it catalyzes the conversion of oxygen ions (O_2_^-^) into oxygen (O_2_) and hydrogen peroxide (H_2_O_2_) (Huang et al., 2016; He et al., 2018), in the AsA-GSH cycle, AsA reduces H_2_O_2_ to water (H_2_O) (Xing et al., 2018). Our findings indicate that bHLH10 may activate stress-responsive gene including APX1 to modulate ROS accumulation under environmental stresses by GRN analysis. Further physiological analyses support this hypothesis, activities of APX increased in transgenic plants under normal, cold and drought stress conditions in our study. In previous study, APX subtypes are present in a variety of organelles as well as in the cytosol, whose adjustment modes differ under environmenta stress conditions (He et al., 2018; Xing et al., 2018). Recent studies shown that *APX1* is induced by heat, cold, drought and H_2_O_2_ stresses, and *APX* genes confer tolerance to various abiotic stresses when overexpressed in transgenic plants (Wang et al., 2016; He et al., 2018; Wang et al., 2020). Absence of cytosolic APX1 in Wassilewskija background results in a breakdown of the H_2_O_2_-scavenging system in *Arabidopsis* chloroplasts, causing an increase in H_2_O_2_ and protein oxidation (Davletova et al., 2005). A knockout of APX1 also significantly inhibits *Arabidopsis* growth and development, leading to increase sensitivity to oxidative stress, stunted growth and delayed flowering (Davletova et al., 2005; Miller et al., 2007; He et al., 2018). In *Populus*, activated PeAPX1 promotes cytosolic APX that scavenges ROS under cold and heat stress, and transgenic *Populus* overexpressing *PtAPX1* showed increased cold tolerance and led to lower MDA and H_2_O_2_ levels in leaf and roots, resulting in higher plant height and root biomass under cold stress, phenocopying the *MrbHLH10* OE plants in our study (He et al., 2018; Zhou et al., 2020; Guo et al., 2021).

**Figure 7:**
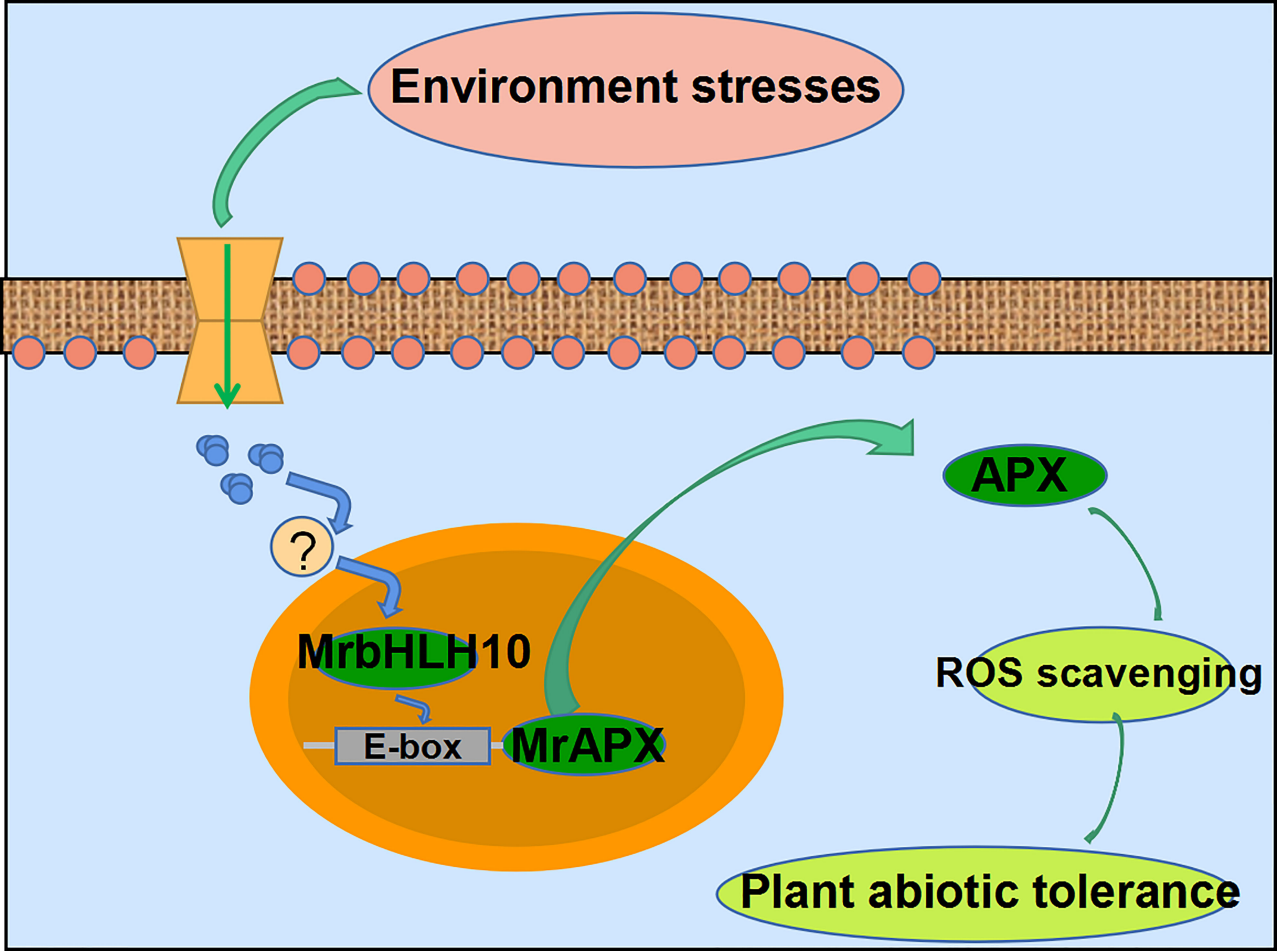
A potential working model for bHLH10 response to abiotic stresses in *M. rubra*. Under drought and cold stress, activating the environmental stresses signaling pathway and stimulating downstream stress signal transduction in plant cells, the upregulated bHLH10 acts upstream of APX and directly regulates its expression by binding to the E-box motif of its promoter. The activated APX then promotes cytosolic ascorbate peroxidase accumulation to scavenge ROS content under environmental stresses.

In this study, we present a systematic review of ROS scavenging regulation first, as well as the complex GRNs that accompany it, and such networks have an essential role to play in understanding plant responses to environmental stresses. Together, the results show that MrbHLH10, a versatile TF, can increase environmental stresses by regulating the expression of MrAPX1, a direct downstream gene, which in turn keeps ROS stable (**Figure 7**). These results indicate that over-expression of bHLH10 can enhance the antioxidative function of transgenic plants, which might be helpful to prevent hyperosmolar and excess ROS due to environmental stresses.

## Data availability statement

Raw data for RNA-seq are available at the BIGD Genome Sequence Archive (https://bigd.big.ac.cn) under accession number CRA006695

## Ethics statement

This study was completed within the laws of the People’s Republic of China. No specific permits were required for our field research. The study species is not included in the ‘List of Protected Plants in China.

## Author contributions

HR and JX designed the conception and experiment; WX, HR, XQ, SZ and ZY performed the experiments; WX wrote the manuscript; JX and HR helped to analyze and assess the data; XQ and SZ provided the valuable suggestions on the manuscript; JX helped to revise the manuscript; HR and JX obtained the funding and were responsible for this manuscript; all authors read and approved the manuscript.
